# Electronic Properties and CO_2_-Selective Adsorption of (NiB)_n_ (*n* = 1~10) Clusters: A Density Functional Theory Study

**DOI:** 10.3390/molecules28145386

**Published:** 2023-07-13

**Authors:** Meiling Hou, Xing Zhou, Chao Fu, Tingting Nie, Yu Meng

**Affiliations:** 1 College of Engineering, Hebei Normal University, Shijiazhuang 050024, China; houml@hebtu.edu.cn (M.H.); zhoux@hebtu.edu.cn (X.Z.); niett@hebtu.edu.cn (T.N.); 2 Hebei Provincial Innovation Center for Wireless Sensor Network Data Application Technology, Shijiazhuang 050024, China; 3 Hebei Provincial Key Laboratory of Information Fusion and Intelligent Control, Shijiazhuang 050024, China; 4 Shaanxi Key Laboratory of Low Metamorphic Coal Clean Utilization, School of Chemistry and Chemical Engineering, Yulin University, Yulin 719000, China; mengyu@yulinu.edu.cn

**Keywords:** (NiB)_n_ clusters, electronic structure, DFT, CO_2_ adsorption

## Abstract

In this study, we investigated the electronic properties and selective adsorption for CO_2_ of nickel boride clusters (NiB)_n_, (n = 1~10) using the first principles method. We optimized the structures of the clusters and analyzed their stability based on binding energy per atom. It was observed that (NiB)_n_ clusters adopt 3D geometries from n = 4, which were more stable compared to the plane clusters. The vertical electron affinity, vertical ionization energy, chemical potential, and highest occupied molecular orbital (HOMO)–lowest unoccupied molecular orbital (LUMO) gap were calculated. Our results revealed that (NiB)_6_ and (NiB)_10_, with high chemical potential, exhibit a higher affinity for CO_2_ adsorption due to a charge delivery channel that forms along the Ni→B→CO_2_ path. Notably, (NiB)_10_ demonstrated a more practical CO_2_ desorption temperature, as well as a broader window for the selective adsorption of CO_2_ over N_2_. The density of states analysis showed that the enhanced CO_2_ adsorption on (NiB)_10_ can be attributed to the synergistic effect between Ni and B, which provides more active sites for CO_2_ adsorption and promotes the electron transfer from the surface to the CO_2_ molecule. Our theoretical results imply that (NiB)_10_ should be a promising candidate for CO_2_ capture.

## 1. Introduction

The issue of global climate change caused by human activities such as fossil fuel consumption and deforestation has become a major concern in the 21st century. The concentration of greenhouse gases, especially CO_2_ [1], has increased dramatically, leading to global warming [2,3]. Although CO_2_ has a lower global warming potential (GWP) than other greenhouse gases like N_2_O and CH_4_, it is thought to considerably contribute to the global greenhouse effect owing to its abundance in the atmosphere. As a result, a significant amount of scientific effort, such as energy efficiency improvement, energy demand reduction, use of renewable energy, electrochemical reduction [4,5], direct hydrogenation of CO_2_ into formic acid, methane, methanol or other chemicals [6,7], and CO_2_ capture and storage [8,9,10], has been concentrated into reducing or controlling the concentration of CO_2_. One of the most important research topics is the separation, storage, and recovery of CO_2_, as it is a reactant in significant industrial processes and a greenhouse gas that contributes to global warming [11]. An ideal CO_2_ sorbent should meet several requirements, including high capacity and selectivity, rapid adsorption/desorption kinetics, and good chemical stability [12].

In recent years, Ni-based materials have received a lot of interest due to their low cost and availability for CO_2_ capture and activity. Roque-Malherbe et al. [13] synthesized Ni–, Zn–, and Cd–nitroprussides, and studied the interactions with CO_2_ molecules. The results showed that Ni–nitroprussides are excellent for CO_2_ storage. However, the accumulation of carbonaceous deposits on the surface of Ni-based catalysts can lead to deactivation [14,15], which eventually blocks the active sites. To address this issue, many attempts have been made with various methods and materials to inhibit the accumulation of carbonaceous deposits [16,17,18,19]. One promising approach is the addition of a small amount of boron to enhance the activity of Ni-based catalysts. First-principle studies suggested that the addition of a small amount of boron can enhance the activity of Ni-based catalysts. Xu and Sayes proposed that boron atoms can effectively block carbon diffusion into nickel lattice by preferentially occupying octahedral sites of the first subsurface layer [20,21]. Experimental studies support this hypothesis. Xu et al. [22] investigated the impact of boron addition on the stability and activity of Ni catalysts used for the steam-reforming of methane. Their results indicate that the catalytic activity of boron-modified catalysts declines with time-on-stream, but less carbon is formed compared to the unpromoted catalyst. Fouskas et al. [17] employed the wet co-impregnation synthesis method to simultaneously deposit Ni and B precursors on the alumina carrier. They demonstrated that B can greatly enhance the resistance to carbon deposition due to its ability to decrease the size of Ni particles. Shakir et al. [23] found that the presence of B facilitated the formation of Ni–B species along with metallic Ni, controlled the particle size and stabilized the metallic state, and influenced the Ni–C interaction, leading to the advancement in catalytic performance and diminution in deactivation.

Ni and B can generate different Ni–B binary compounds. Shein et al. [24] calculated the lattice constant, magnetic properties, and formation energy of Ni_3_B, which indicated that the strong stability of Ni_3_B was due to the strong Ni–B hybridization. Zhou et al. [25] researched the electronic structure and mechanical properties of NiB. However, there is no literature on the structure and electronic property of the NiB cluster and its availability for CO_2_ capture and activity.

To summarize, the development of efficient and effective CO_2_ sorbents is crucial for reducing the concentration of greenhouse gases and mitigating the effects of global climate change. Ni-based materials, especially those modified with boron, have shown great potential for CO_2_ capture and activity. In our research, we conducted a comprehensive investigation on the structures, electronic properties, CO_2_ adsorption, and activities of NiB clusters of various sizes using first-principles calculations. The obtained results could provide guidance for the properties of Ni-based material for CO_2_ adsorption and activity, which could play a crucial role in mitigating the effects of global climate change.

## 2. Results

### 2.1. Geometrical Structures of Clusters

**Figure 1:**
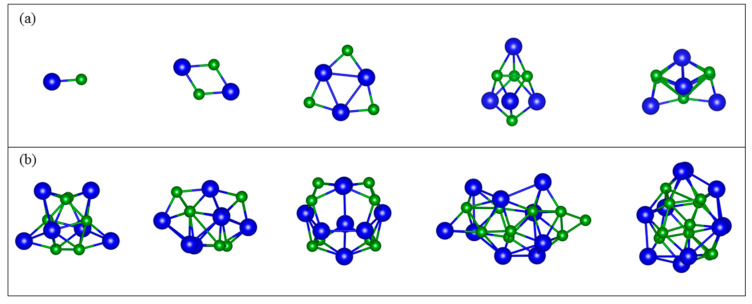
Ground-state (NiB)_n_ cluster structures depicting the geometry evolution from linear (*n* = 1), to planar (*n* = 2, 3), to 3D (*n* = 4~10). (a): *n* = 1~5; (b): *n* = 6~10. Blue and green spheres depict Ni and B atoms, respectively.

To begin, we constructed and optimized the lowest-energy structures of the most stable (NiB)_n_ (*n* = 1~10) clusters. The resulting ground states of the (NiB)_n_ (*n* = 1~10) are visible in Figure 1. For (NiB)_1_, the ground-state structure is linear. The bond length and the binding energy for NiB dimer is 1.68 Å and −7.13 eV/atom, respectively. For (NiB)_2_, a rhombus structure with four bonds of 1.81 Å is obtained as the ground-state structure. In the case of *n* = 3, it is observed that the planar structure exhibits an inner Ni atom triangle, which is nearly equilateral, with a bond length of 1.80 Å. For *n* = 4–10, (NiB)_n_ clusters adopt 3D configurations, with a bond of ca. 1.95 Å. Clusters (*n* = 4, 5, 6, 8) are especially the most symmetric.

**Figure 2:**
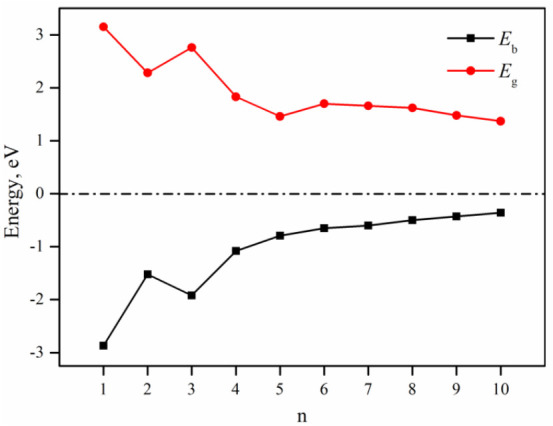
Binding energy (*E*_b_, in eV) and the highest occupied molecular orbital (HOMO)–lowest unoccupied molecular orbital (LUMO) gap (*E*_g_) of (NiB)_n_ (*n* = 1–10) clusters.

Next, we turn to the electronic properties of these (NiB)_n_ clusters. The data reported in Figure 2 show that the binding energy per atom (*E*_b_) exhibits an asymptotic behavior. In this figure, it is observed that beyond the 8-atom cluster (*n* = 4), the value of Eb increases slowly as the cluster size increases. The exponential fitting for (NiB)_n_ (*n* = 4~10) gives $E_{b}=0.3557\ln\left(n\right)-1.065$, and R^2^ value of 0.994. This trend is rationalized by noticing that the average number of nearest neighbors grows with cluster size, thus increasing the number of atomic interactions [26]. Remarkably, this nuclearity represents the size after which the clusters change from planar to 3D. Therefore, Figure 2 depicts the stabilization toward 3D structures [27].

In addition to the findings discussed in the previous paragraph, the study also calculated the VIE and VEA for each size cluster (Table 1). The results show that the (NiB)_n_ cluster has a tendency to donate an electron, as indicated by the positive VIE and VEA [28,29]. The VIE and VEA values were also used to determine the global reactivity descriptors (GRDs) [30,31], with the most widely used parameter being μ. The μ value reflects the escaping tendency of an electron, with higher values indicating greater reactivity [30]. The low μ values of (NiB)_n_ (*n* = 1–3) clusters indicate their low reactivity. This is consistent with their low chemical potential. Additionally, these cluster sizes exhibit high band gap energies (*E*_g_), as shown in Figure 2, while for (NiB)_n_ clusters with n > 4, the band gap energies are lower. Consequently, these clusters may exhibit higher reactivity compared to (NiB)_n_ (*n* = 1–3) clusters. The band gap reflects the possibility of electrons to jump from occupied to unoccupied orbitals. Based on the high *E*_g_ and low μ value, clusters *n* = 1–3 are identified as least reactive in the series presented here. Therefore, this paper only focuses on the (NiB)_n_ structures *n* = 4–10.

### 2.2. CO_2_ Adsorption

**Figure 3:**
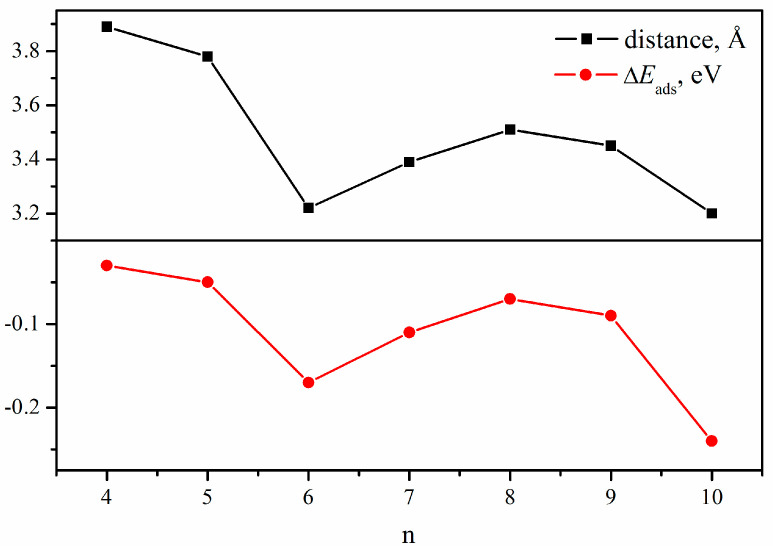
The distance and adsorption energy of CO_2_ adsorption on (NiB)_n_ clusters.

**Figure 4:**
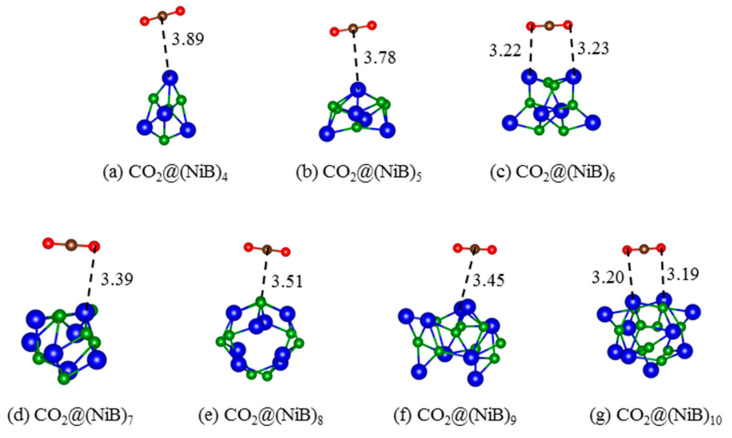
Relaxed local geometric structures for CO_2_ adsorbed on (NiB)_n_. Brown and red spheres depict C and O atoms, respectively. All lengths are given in Å.

This passage focuses on the physisorption of CO_2_ molecules on different (NiB)_n_ surfaces. The initial placement of all CO_2_ molecules was at a distance of 2.5 Å above the surface, to simulate the physisorption process. As shown in Figure 3, (NiB)_10_ has the highest chemical potential and possesses the highest activity for CO_2_ adsorption compared to all the structures studied, followed by (NiB)_6_. Furthermore, it is also found that the distance between CO_2_ and the surface (Figure 3) is highly correlated with the adsorption energies. CO_2_ tends to adsorb at the top of Ni instead of B (Figure 4), except adsorption on (NiB)_8_, which illustrates that CO_2_ should preferably obtain electrons from the Ni atoms rather than B atoms in the cluster. For CO_2_ adsorption on (NiB)_8_, CO_2_ binds to B instead of Ni due to the spherical-like structure of (NiB)_8_, which creates a more exposed or accessible surface for B atoms at the top.

**Figure 5:**
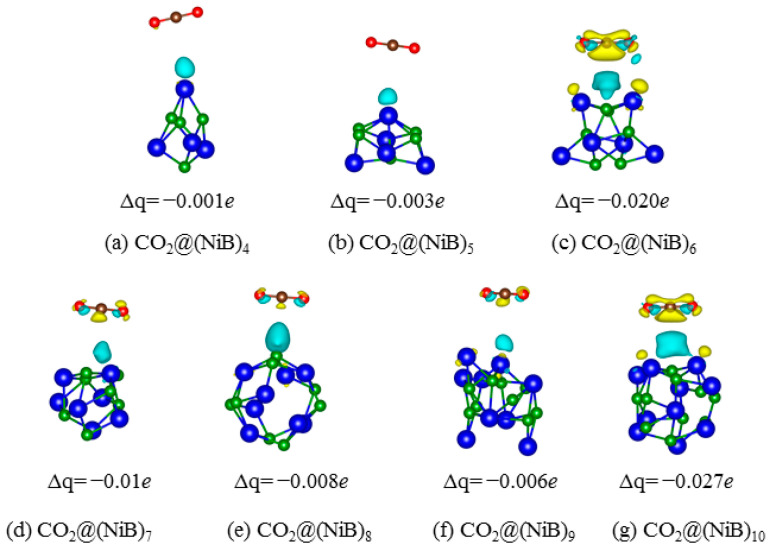
Charge difference of the adsorption of CO_2_ on (NiB)_n_. The yellow cloud indicates electron depletion, while the blue indicates accumulation. The iso-surface is 0.0003 e/Å^3^. Δq stands for the total charge of CO_2_ molecule and is given in e. Negative means charge obtainment.

The local structures and charge differences for different adsorption complexes are shown in Figure 5. Compared with other complexes, CO_2_ molecules obtain additional electron from the unsaturated Ni atom of (NiB)_6_ and (NiB)_10_ (Figure 5c,g). Thus, the adsorption enhancement by the two surfaces is reasonable. Most importantly, (NiB)_10_ is determined to be the most active for CO_2_ adsorption by its largest Δ*E*_ads_ (−0.24 eV) and with the most charges transferred to CO_2_ (Δq = −0.027 *e*). As per Figure 5g, a charge delivery channel forms along the Ni→B→CO_2_ path, which provides more electrons traveling from the surface to CO_2_ molecules. The channel is strengthened in the order of (NiB)_4_ < (NiB)_5_ <(NiB)_8_ < (NiB)_9_ < (NiB)_7_ < (NiB)_6_ < (NiB)_10_, in accordance with the adsorption energy. This suggests that the charge delivery channel along the Ni→B→CO_2_ path is the key factor that contributes to the strong adsorption capacity of (NiB)_10_ for CO_2_. The charge delivery channel provides a pathway for the transfer of electrons from the cluster to the CO_2_ molecule, which enhances the CO_2_ adsorption. The results suggest that the synergistic effect between Ni and B provides more active sites for CO_2_ adsorption and promotes the electron transfer from the surface to the CO_2_ molecule. The larger cluster size leads to a higher adsorption energy for CO_2_, which is attributed to the formation of the charge delivery channel along the Ni→B→CO_2_ path. These findings have important implications for the design of efficient sorbents for CO_2_ capture and separation, which is crucial for mitigating the negative impacts of greenhouse gas emissions on the environment.

### 2.3. Adsorption Selectivity over N_2_

**Figure 6:**
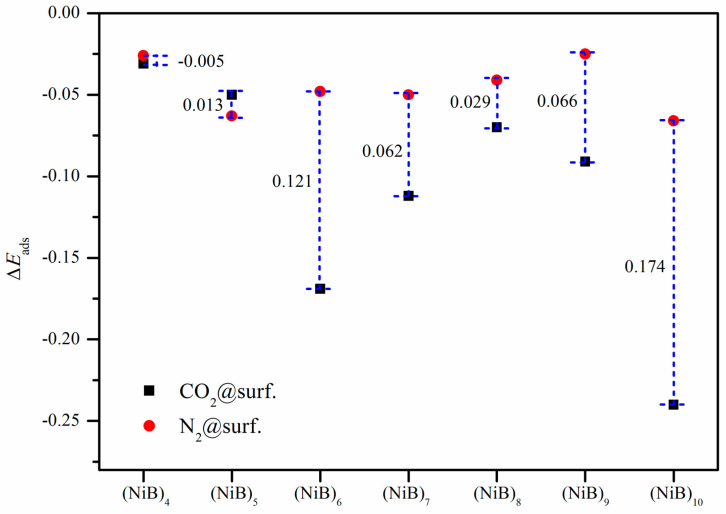
Selective adsorption of CO_2_ over N_2_ on different (NiB)_n_ surfaces. Black squares and magenta circles refer to the Δ*E*_ads_ CO_2_ and N_2_ adsorption, respectively.

In addition to the remarkable CO_2_ adsorption activity, an effective adsorbent should control selectivity over other competitive gaseous molecules, especially N_2_. Hence, N_2_ adsorption on different (NiB)_n_ surfaces was also studied and the corresponding results are listed in Figure 6. Interestingly, the largest adsorption enhancement of N_2_ is still located in the (NiB)_10_ slab of −0.066 eV, which has a similar pattern to CO_2_ adsorption.

Subsequently, CO_2_ adsorption selectivity over N_2_ is identified in Figure 6. An ignorable distinction in the calculated results is discovered between CO_2_ and N_2_ absorbed on (NiB)_4_, (NiB)_5_, and (NiB)_8_. However, the two widest variations occur in the situations of CO_2_ adsorption on (NiB)_6_ and (NiB)_10_ with the largest increase in adsorption energy. Taking CO_2_@(NiB)_10_ as an example, the Δ*E*_ads_ (−0.24 eV) is almost four times that for N_2_ (−0.066 eV) on the same surface, which shows that (NiB)_10_ exhibits the potency of a highly selective adsorbent for CO_2_ capture.

The results suggest that the larger cluster size leads to a higher adsorption energy for both CO_2_ and N_2_, but the increase in adsorption energy for CO_2_ is more significant than that for N_2_. The results indicate that (NiB)_n_ clusters have the potential to be highly selective adsorbents for CO_2_ capture and separation, which is crucial for the development of sustainable energy technologies.

**Figure 7:**
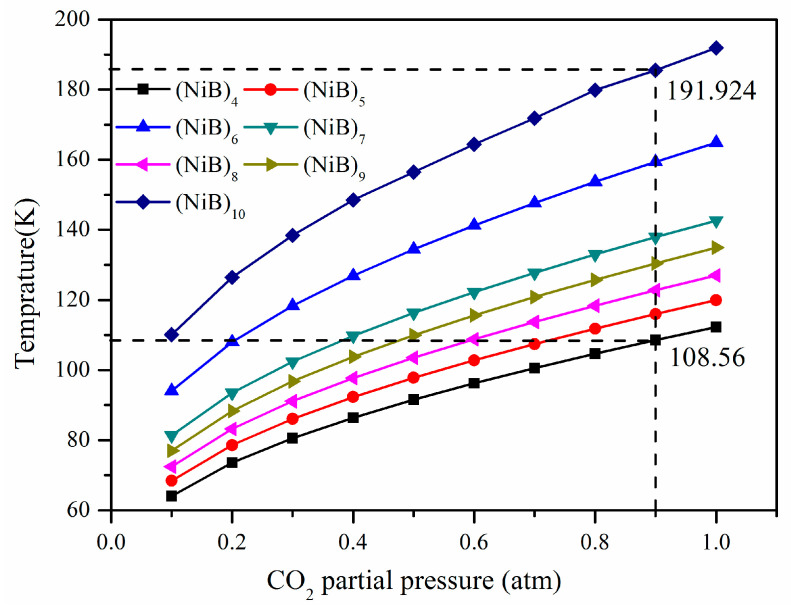
T–P phase diagram of CO_2_ adsorption on (NiB)_n_ (*n* = 4–10).

**Figure 8:**
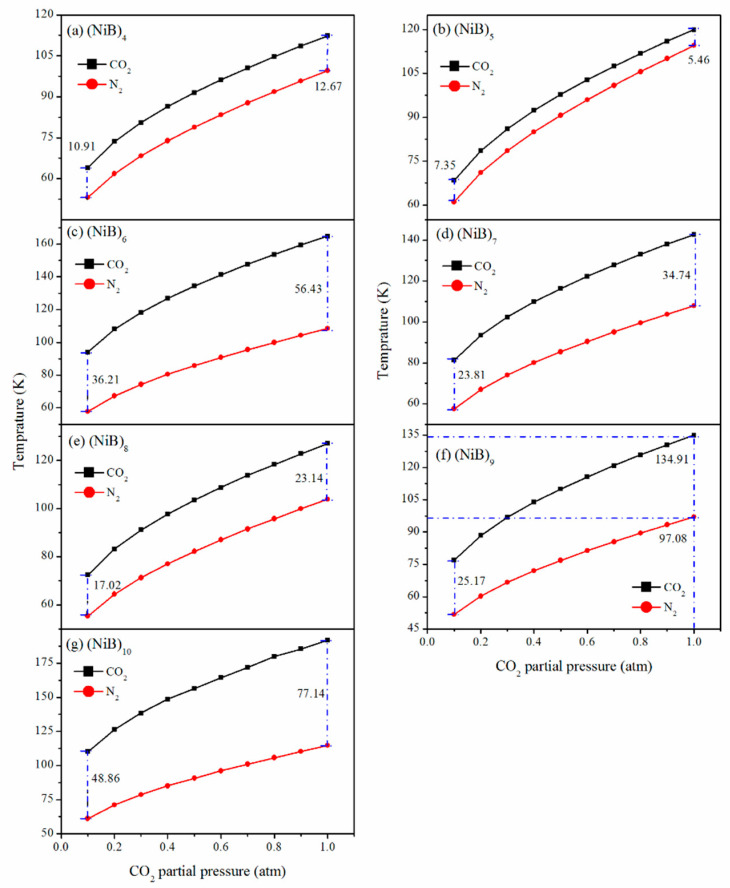
T–P phase diagram of the CO_2_ adsorption over N_2_ on (NiB)_n_.

Strictly, the adsorption energies calculated for CO_2_ and N_2_ on (NiB)_n_ (*n* = 4–10) slabs, as discussed earlier, have the Helmholtz-free energy of 0 k, without zero-point energy correlation. To evaluate the relative adsorption stability for different configuration species under finite temperature and pressure, we adopted the ab initio thermodynamic approach (Equation (7)) and extended our DFT energy results to plot the temperature and pressure (T–P) phase diagram between CO_2_@surfaces and N_2_@surfaces in the temperature and pressure range of our interest (Figure 7 and Figure 8).

For example, taking 0.9 atm as the CO_2_ partial pressure, for example, in Figure 7, the desorption temperature is raised in the order of CO_2_@(NiB)_4_ < CO_2_@(NiB)_5_ < CO_2_@(NiB)_8_ < CO_2_@(NiB)_9_ < CO_2_@(NiB)_7_ < CO_2_@(NiB)_6_ < CO_2_@(NiB)_10_. The desorption temperature is raised from 108.56 K for CO_2_@(NiB)_4_ to 191.924 K for CO_2_ (NiB)_10_. This means the application range for CO_2_ adsorption is extended to about 80 K by larger cluster size, leading to a superior adsorbent for CO_2_ capture under mild conditions.

The T–P phase diagram provides a comprehensive understanding of the adsorption behavior of CO_2_ and N_2_ on (NiB)_n_ clusters. It shows that the adsorption selectivity of CO_2_ over N_2_ is highly dependent on the cluster size and structure, and the larger cluster size leads to a higher desorption temperature for CO_2_. The results suggest that (NiB)_n_ clusters can be a promising candidate for CO_2_ capture and separation under mild conditions, which is crucial for the development of sustainable energy technologies.

Figure 8 illustrates the equilibrium states for the adsorption of a single gas species under varying temperature and pressure conditions. The curves in the graph stand for the adsorption isotherms for each gas species. In the upper left part above the curve, the CO_2_/N_2_ molecule is inclined to be desorbed as a gaseous species, while the bottom right part indicates an adsorbed state. The interval between two different curves is positively correlated with their selectivity over each other.

For instance, taking the CO_2_/N_2_ adsorption on (NiB)_6_ as an example (Figure 8c), under the partial pressure of 1.0 atm, N_2_ can be adsorbed below 97.08 K and desorbed above this temperature. However, the desorption temperature of CO_2_ is 134.91 K. Therefore, in the temperature range of 97.08–134.91 K, (NiB)_9_ selectively adsorbs CO_2_ over N_2_. The temperature range can be defined as the selective window of CO_2_/N_2_. Compared with the situation of (NiB)_6_ and (NiB)_10_, the selective window for CO_2_/N_2_ is distinctly narrowed on five other surfaces. The results suggest that the selectivity of CO_2_/N_2_ adsorption on NiB clusters is highly dependent on the cluster size and structure. These findings provide valuable insights into the design of efficient sorbents for CO_2_ capture and separation.

In addition, the results suggest that the selectivity of CO_2_/N_2_ adsorption on NiB clusters is highly dependent on the cluster size and structure. The (NiB)_n_ cluster with a higher chemical potential can greatly elevate the desorption temperature of various gases, making it more practical for industrial use. These findings provide valuable insights into the design of efficient sorbents for CO_2_ capture and separation, which is crucial for mitigating the negative impacts of greenhouse gas emissions on the environment.

## 3. Discussion

**Figure 9:**
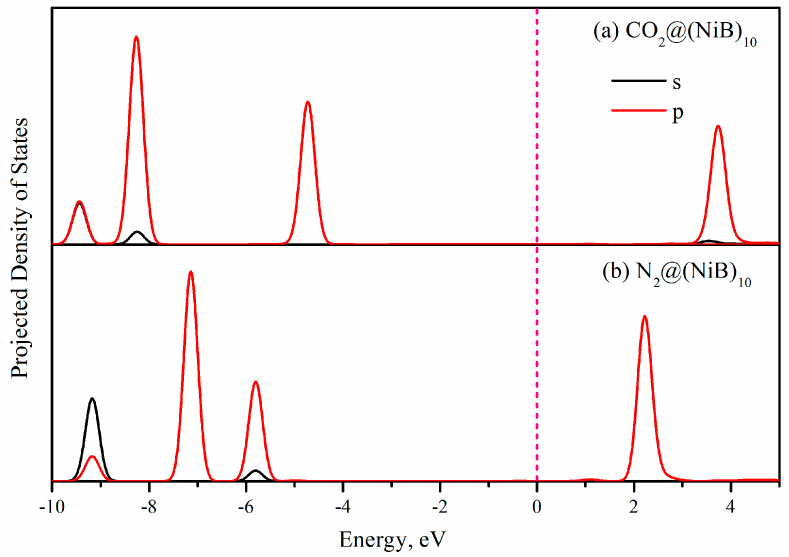
Projected density of states (PDOS) analysis for the adsorption of CO_2_ and N_2_ on the (NiB)_10_ surface.

For the adsorption of CO_2_ and N_2_ on (NiB)_10_, the PDOS (projected density of states) have been calculated and shown in Figure 9. It was observed that the DOS of CO_2_ is closer to the Fermi level compared to that of N_2_. In the case of CO_2_, its DOS being closer to the Fermi level suggests a higher availability of energy states for CO_2_ molecules to interact with the (NiB)_10_ surface. This proximity of energy levels facilitates stronger interactions between CO_2_ and the adsorbent, leading to a higher adsorption affinity. On the other hand, N_2_, with its DOS farther away from the Fermi level, has fewer available energy states for interaction, resulting in weaker adsorption.

It has been recognized that the Ni→B→CO_2_ path provides more electrons traveling from the surface to the CO_2_ molecule, which enhances the CO_2_ reduction activity. In this study, we further discuss this point and make some generalizations of our findings.

**Figure 10:**
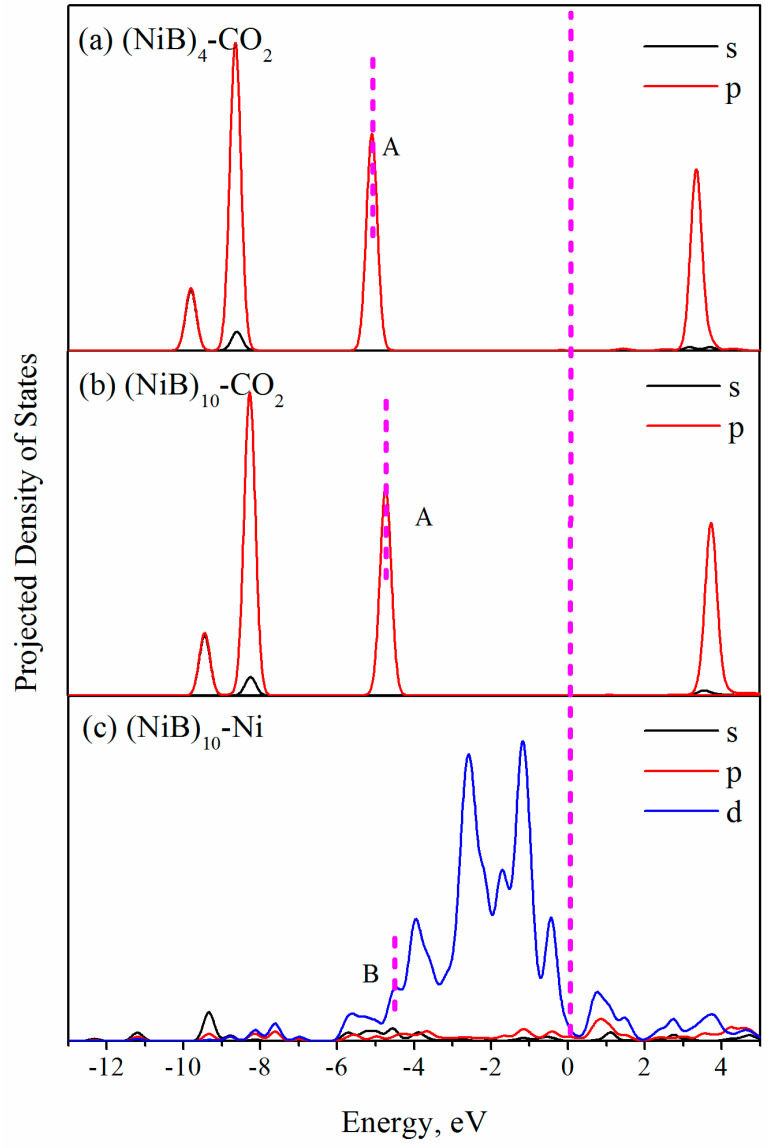
Projected density of states for CO_2_ on (NiB)_4_ and (NiB)_10_ (**a**,**b**). (**c**) is the Ni atom close to CO_2_ in (NiB)_10_. The Fermi level is set to 0 eV.

To investigate the CO_2_ adsorption on the (NiB)_n_ cluster, we calculated the projected density of states (PDOS) for the adsorption of CO_2_ on (NiB)_4_ and (NiB)_10_, which exhibited the lowest and largest adsorption energies, respectively. Figure 10 displays the results of this analysis. Compared with CO_2_ on (NiB)_4_ (Figure 10a), the localized CO_2_ 2p peak A was enhanced and shifted to a higher energy level. This indicates that the CO_2_ molecule is strongly adsorbed on the (NiB)_10_ surface, which is consistent with the high adsorption energy. Furthermore, Ni atoms in clusters hybridized with CO_2_ (peak B in Figure 10c) and should be polarized with the approach of positively charged species (e.g., C atom in CO_2_ molecule), which is consistent with the formation of the charge delivery channel along the Ni→B→CO_2_ path.

This suggested that the charge delivery channel and the hybridization of Ni atoms with CO_2_ are the key factors that contribute to the strong CO_2_ adsorption capacity of (NiB)_10_. The charge delivery channel provides a pathway for the transfer of electrons from the cluster to the CO_2_ molecule, while the hybridization of Ni atoms with CO_2_ enhanced the interaction between the cluster and the CO_2_ molecule. The polarization of Ni atoms in a positively charged manner further strengthens the interaction between the cluster and the CO_2_ molecule, which increases the adsorption capacity of CO_2_ on the cluster surface. This suggests that the CO_2_ adsorption promotion by (NiB)_10_ should be predictable. The enhanced CO_2_ adsorption on (NiB)_10_ can be attributed to the synergistic effect between Ni and B, which provides more active sites for CO_2_ adsorption and promotes electron transfer from the surface to the CO_2_ molecule.

In conclusion, our study provides a deeper understanding of the CO_2_ adsorption on NiB and sheds light on the design of efficient sorbents for CO_2_ capture. The results suggest that NiB is a promising sorbent for CO_2_ molecules due to its high activity and selectivity, and the Ni→B→CO_2_ path provides a promising strategy to enhance the CO_2_ adsorption performance.

## 4. Calculation Methods

All the clusters were optimized with the Vienna Ab initio Simulation Package (VASP5.4) [32,33], which uses a plane wave basis set to solve the Kohn–Sham equation of the density functional theory (DFT). The Perdew–Burke–Ernzerhof (PBE) exchange correlation function [34] was used, and the cut-off energy was set to 450 eV for all the calculations within the framework of the projector-augmented wave (PAW) method [35,36]. The (NiB)_n_ clusters were placed in a 30 × 30 × 30 Å cubic super-cell, which makes the interaction between the two neighboring cluster images negligible. The Brillouin zone was sampled with 5 × 5 × 1 k-points sampled using the Monkhorst–Pack method. The cluster structures were converged when the force components were less than a threshold value of 0.02 eV Å^−^^1^, and the change of total energy less than 10^−^^5^ eV. The frequency calculations were performed to confirm that the reported clusters are geometrically stable, and at the same time, to get the zero-point vibration energies (ZPE) of the clusters. If not mentioned, the ZPE correction was included in the total energy.

The binding energy per atom (*E*_b_) for the most stable structures of (NiB)_n_ clusters are calculated in order to predict the relative stability of the clusters. The binding energy per atom (*E*_b_) was estimated through Equation (1):

*E*_b_ = −[*E*_cluster_ − n(*E*_Ni_ + *E*_B_)]/N (1)

where N = 2n is the total number of atoms in the cluster. The total energy, *E*_cluster_, corresponds to the lowest energy obtained for a set of same-sized clusters, while *E*_Ni_ and *E*_B_ stand for the total ground state energy of Ni and B atoms, respectively.

Next, we calculated the vertical electron affinity (VEA), vertical ionization energy (VIE), chemical potential (μ), and highest occupied molecular orbital (HOMO)–lowest unoccupied molecular orbital (LUMO) gap (*E*_g_) values according to the formulae below:

VEA = *E*_neutral_ − *E*_anion_ (2)

VIE = *E*_cation_ − *E*_neutral_ (3)

μ = −1/2(VIE + VEA)(4)

*E*_g_ = *E*_LUMO_ − *E*_HOMO_ (5)

The adsorption energy (Δ*E*_ads_) of (NiB)_n_ toward CO_2_ and N_2_ is defined as:

Δ*E*_ads_ = *E*_tot_ − (*E*_mol_ + *E*_sheet_) (6)

where *E*_tot_, *E*_mol_, and *E*_sheet_ are the total energies of the adsorption complex, the isolated molecule, and the (NiB)_n_, respectively.

The ab initio thermodynamic method [37] is used to calculate the Gibbs free energy G(T, P) of the adsorption processes by taking environmental effects into account. Due to the variation in solid-phase adsorbents under different temperatures and pressures being negligible [38,39], the adsorption of CO_2_ gas is regarded as the main contributor leading to the Gibbs free energy change. The formula for calculation is given below:

(7) $$\Delta G_{ads}(T,P)=E_{ads}^{DFT}-E_{slab}^{DFT}-E_{g}^{DFT}+\Delta F^{vib,ads}(T)-[\Delta H_{g}(0K\to T,P^{0})-TS_{g}(T,P^{0})+kT\ln(\frac{P}{P^{0}})$$

where $F^{vib,ads}(T)$ means the zero-point energy (ZPE) correction, which is considered in all adsorption processes of gases. The contribution of gas enthalpy and entropy under atmospheric pressure (P^0^ = 1 × 10^5^ Pa) can be obtained in the thermodynamic database.

For the adsorbed product CO_2_@(NiB)_n_ and the corresponding free molecule in the gaseous phase, the vibrational contributions from n prominent vibrational modes within the harmonic approximation to the Gibbs free energy (comprising vibrational energy and entropy) are defined as:

(8) $$F^{vib}\left(T\right)=\sum_{i=1}^{n}F^{vib}(T,v_{i})=\sum_{i=1}^{n}[\frac{hv_{i}}{2}+kT\ln(1-e^{-\beta hv_{i}})]$$

where β = 1/kT and vi are the vibration frequency. In our cases, the total vibrational correction can be expressed as:

(9) $$F^{vib,ads}\left(T\right)=F_{ads}^{vib}\left(T\right)-F_{g}^{vib}\left(T\right)$$

where $F_{ads}^{vib}\left(T\right)$, $F_{g}^{vib}\left(T\right)$, and $F^{vib,ads}\left(T\right)$ are vibrational Gibbs free energies of the adsorbed molecule, the free gas molecule, and their change arising from the adsorbate–surface interaction, respectively.

The charge density difference for the gas-interacted (NiB)_n_ is plotted to understand the nature of bonding between the gases and the (NiB)_n_ sheet. The charge density difference plot is calculated as follows:

(10) $$\Delta \rho =\rho^{complex}-\underset{i}{\sum }\rho_{i}^{fragments}$$

where $\rho^{complex}$ is the density of the complex, and $\rho_{i}^{fragments}$ is the density of the fragments forming the whole system, where fragments consist of (NiB)_n_ and gas molecules. The electronic properties of density of states (DOS) was also evaluated to better understand the nature of chemical bonding.

## 5. Conclusions

The adsorption of carbon dioxide (CO_2_) has become a crucial research topic in recent years due to the increasing concerns about global warming and climate change. The capture and storage of CO_2_ is a promising method, which has sparked a lot of scientific interest. The development of efficient sorbents for CO_2_ capture and storage is crucial to enhance the performance of the electrochemical process.

In this study, we employed first-principles calculations to investigate the stable structures of (NiB)_n_ clusters (n = 1–10) and their interaction with CO_2_. We first located the structures of (NiB)_n_ clusters and found that they adopt 3D geometries from n = 4. The calculation of binding energy per atom (E_b_) revealed that 3D geometries were more stable than plane clusters. We discussed the chemical reactivity of each atom in the clusters based on the calculated vertical electron affinity, vertical ionization energy, chemical potential, and highest occupied molecular orbital (HOMO)–lowest unoccupied molecular orbital (LUMO) gap. Our analysis showed that (NiB)_6_ and (NiB)_10_ with high chemical potential may be more favorable for CO_2_ adsorption.

We further investigated the adsorption of CO_2_ on various clusters and the selective adsorption of N_2_ among these clusters. Our results substantiated that (NiB)_6_ and (NiB)_10_ exhibited the strongest adsorption of CO_2_ due to a charge delivery channel that forms along the Ni→B→CO_2_ path, which allows for more electrons traveling from the surface to CO_2_ molecules. The results of the density of states analysis show that Ni atoms in clusters hybridized with CO_2_ and should be polarized with the approach of positively charged species. In particular, (NiB)_10_ showed the highest adsorption selectivity of CO_2_ over N_2_, followed by (NiB)_6_. Notably, we found that (NiB)_10_ had a more practical CO_2_ desorption temperature and a broader window for the selective adsorption of CO_2_ over N_2_.

Our theoretical results suggest that (NiB)_10_ could be a promising candidate for CO_2_ capture. Moreover, the (NiB)_n_ cluster with a higher chemical potential can significantly increase the desorption temperature of various gases, making it more practical for industrial use.

## Figures and Tables

**Table 1:** Vertical ionization energy (VIE), vertical electron affinity (VEA), and chemical potential (μ) of (NiB)_n_ (*n* = 1–10) clusters (units are in eV).

Cluster Form	VIE	VEA	μ
(NiB)_1_	5.22	2.84	−4.03
(NiB)_2_	5.58	2.43	−4.01
(NiB)_3_	4.84	3.56	−4.20
(NiB)_4_	3.85	2.56	−3.21
(NiB)_5_	3.40	2.49	−2.94
(NiB)_6_	3.21	2.41	−2.81
(NiB)_7_	3.37	2.67	−3.02
(NiB)_8_	4.52	1.77	−3.18
(NiB)_9_	5.17	1.29	−3.23
(NiB)_10_	2.15	2.07	−2.11

## Data Availability

Data will be available upon request via email to the corresponding authors.

## Sample Availability

Samples of the compounds are not available from the authors.
